# Skin-homing basophils and beyond

**DOI:** 10.3389/fimmu.2022.1059098

**Published:** 2022-12-22

**Authors:** Rintaro Shibuya, Brian S. Kim

**Affiliations:** ^1^ Kimberly and Eric J. Waldman Department of Dermatology, Icahn School of Medicine at Mount Sinai, New York City, NY, United States; ^2^ Mark Lebwohl Center for Neuroinflammation and Sensation, Icahn School of Medicine at Mount Sinai, New York City, NY, United States; ^3^ Department of Dermatology, Kyoto University Graduate School of Medicine, Kyoto, Japan; ^4^ Marc and Jennifer Lipschultz Precision Immunology Institute, Icahn School of Medicine at Mount Sinai, New York City, NY, United States; ^5^ Friedman Brain Institute, Icahn School of Medicine at Mount Sinai, New York City, NY, United States

**Keywords:** basophil, CysLTR2, histamine, IL-33 and ST2, leukotriene C4, mast cell, MRGPRX2

## Abstract

Basophils have been implicated in type 2 inflammation and numerous disorders in the skin such as helminth infection, atopic dermatitis, and urticaria. Although similar in form and function to tissue-resident mast cells, classical studies on basophils have centered on those from the hematopoietic compartment. However, increasing studies in tissues like the skin demonstrate that basophils may take on particular characteristics by responding to unique developmental, chemotactic, and activation cues. Herein, we highlight how recent studies in barrier immunology suggest the presence of skin-homing basophils that harbor a unique identity in terms of phenotype, function, and motility. These concepts may uniquely inform how basophils contribute to diseases at multiple epithelial surfaces and our ability to therapeutically target the innate immune system in disease.

## Introduction

Basophils are rare granulocytes, accounting for <1% of leukocytes in the peripheral blood, spleen, and bone marrow. Basophils were first described by Paul Ehrlich in 1879. Subsequently, several groups have discovered that basophils in the blood are a source of histamine in the 1950s (1–3). However, it was not until 1972 that basophils were shown to be activated by allergens in an IgE-mediated fashion (4). Given their similarity in form and function to tissue-resident mast cells, basophils have long been considered “circulating mast cells”, although their differences and similarities are often debated. Thus, they have long been studied as a surrogate for mast cells due to their accessibility *via* the peripheral blood.

Monitoring of human basophils by flow cytometry has revealed changes in cell surface markers and activation of basophils (5). Moreover, a recent study on human basophils by Blom et al. reported unique chemokine receptor expression patterns upon IgE-mediated or non-IgE-mediated activation, strongly suggesting heterogeneous activation manners in human basophils (6). In contrast to human basophils, the characteristics and functions of murine basophils *in vivo* have come to light with the advent of antibody-mediated cell depletion methods (e.g., anti-FcϵRIa, CD200R3, or Thy1 antibodies) (7–9). However, such methods were not sufficient to distinguish the unique role of murine basophils from mast cells *in vivo* due to the bystander effects on mast cells. This problem was overcome with the development of unique transgenic mouse technologies and the identification of basophil-specific genes and markers (e.g., Mcpt8-DTR, Mcpt8-Cre, Bas-TRECK Tg, and Basoph8 mice) (10–13). Indeed, these advances have made it possible to directly compare basophils with mast cells, revealing that these two myeloid cell populations differ in surface marker expression, factors required for terminal differentiation, signaling pathways, release of inflammatory mediators, and impact on disease.

Furthermore, it is generally accepted that basophils are effector cells of the innate immune system that promote type 2 immunity and inflammation through the release of a variety of mediators including the type 2 cytokines IL-4 and IL-13. Although residing in the circulation, basophils are rapidly recruited into the tissues such as the intestine, lung, and skin upon inflammation (14). Thus, they have been implicated in promoting the expulsion of helminth parasites from mucosal barriers and in the pathophysiology of a variety of allergic disorders such as asthma, atopic dermatitis (AD), food allergy, and chronic spontaneous urticaria (CSU) (15–19). Further, recent studies have shed light on novel functions of basophils which may even reside in peripheral organs (20–22). However, how basophils are recruited to the tissues upon stimulation and the manner in which they are activated or survive in tissues remain poorly understood. Moreover, the precise contribution of basophils to various allergic disorders such as AD continues to be debated even though many studies have implicated basophils as putative drivers in AD pathogenesis based on both mouse and human studies (17, 23–27).

Herein, we highlight recent advances in basophil biology in peripheral organs such as the skin and how they provoke new hypotheses and theories about basophil function more broadly. We propose revisiting a number of assumptions about the properties of basophils in tissues using new approaches, technology, and therapeutics.

## Developmental, maturation, and activation cues from the tissue

Both basophils and mast cells differentiate from hematopoietic stem cells *via* common myeloid progenitors and granulocyte monocyte progenitors (GMPs). Although similar in terms of granularity, expression of the high affinity IgE receptor (FcϵRI), and shared effector molecules, basophils largely reside in the circulation while mast cells reside in other tissues. Recent studies demonstrate that mast cells arise from the yolk sac and aorta-gonad-mesonephros, and the degree to which they are replenished by bone marrow precursors is variable depending on the organ (28). Skin-resident mast cells, in particular, are devoid of bone marrow-derived mast cells and are mostly seeded in the early phase of embryonic development (29, 30). These findings help to explain, at least in part, why the majority of allergic disorders involving mast cells develop early in life. Furthermore, these findings provoke the hypothesis that dysregulated mast cell development could be one explanation for the heterogeneity of allergic pathologies and therapeutic responses. Notwithstanding these insights into the diversity of mast cell subpopulations, it is largely unknown whether related developmental sophistication underlies basophil heterogeneity.

IL-3 is an important growth factor for both basophils and mast cells (31). For example, IL-3 deficient mice exhibited impaired expansion of basophils and mast cells in a setting of nematode infection despite no obvious abnormality in their number at steady state (32). IL-3 is also capable of promoting basophil differentiation from bone marrow cells and survival *in vitro* (33, 34). Moreover, IL-3 augments cytokine production from basophils after IgE crosslinking, a canonical activation mechanism in basophils (35). Collectively, many of these early studies established IL-3 as a key regulatory cytokine for basophils as well as mast cell proliferation and function. However, most of these studies centered on studying basophils within the hematopoietic compartment. The precise properties of basophils within barrier tissues have been traditionally poorly understood.

In addition to IL-3, granulocyte–macrophage colony-stimulating factor (GM-CSF), Toll-like receptors (TLRs), and thymic stromal lymphopoietin (TSLP) are also known to regulate basophil development (36–38). Among them, TSLP has been shown to act directly on bone marrow and extramedullary progenitors to promote basophil hematopoiesis independently of IL-3 in mice (20, 36). Furthermore, murine basophils differentiated by TSLP have unique transcriptional profiles and activation states compared to those developed under IL-3-enriched conditions (20). In contrast to murine basophils, human basophils from healthy donors do not respond to TSLP without IL-3 priming (39). However, disease-associated human basophils from patients with asthma were responsive to TSLP alone (40). These findings suggest that inflammatory conditions could affect the responsiveness of the human basophil. In the skin, TSLP is consistently upregulated during AD-associated skin inflammation and has long been pursued as a therapeutic target in humans (41, 42). However, the efficacy of targeting TSLP as a therapy in AD has been brought into question, as the anti-TSLP monoclonal antibody (mAb) tezepelumab has not been successful in treating AD (43).

**Figure 1:**
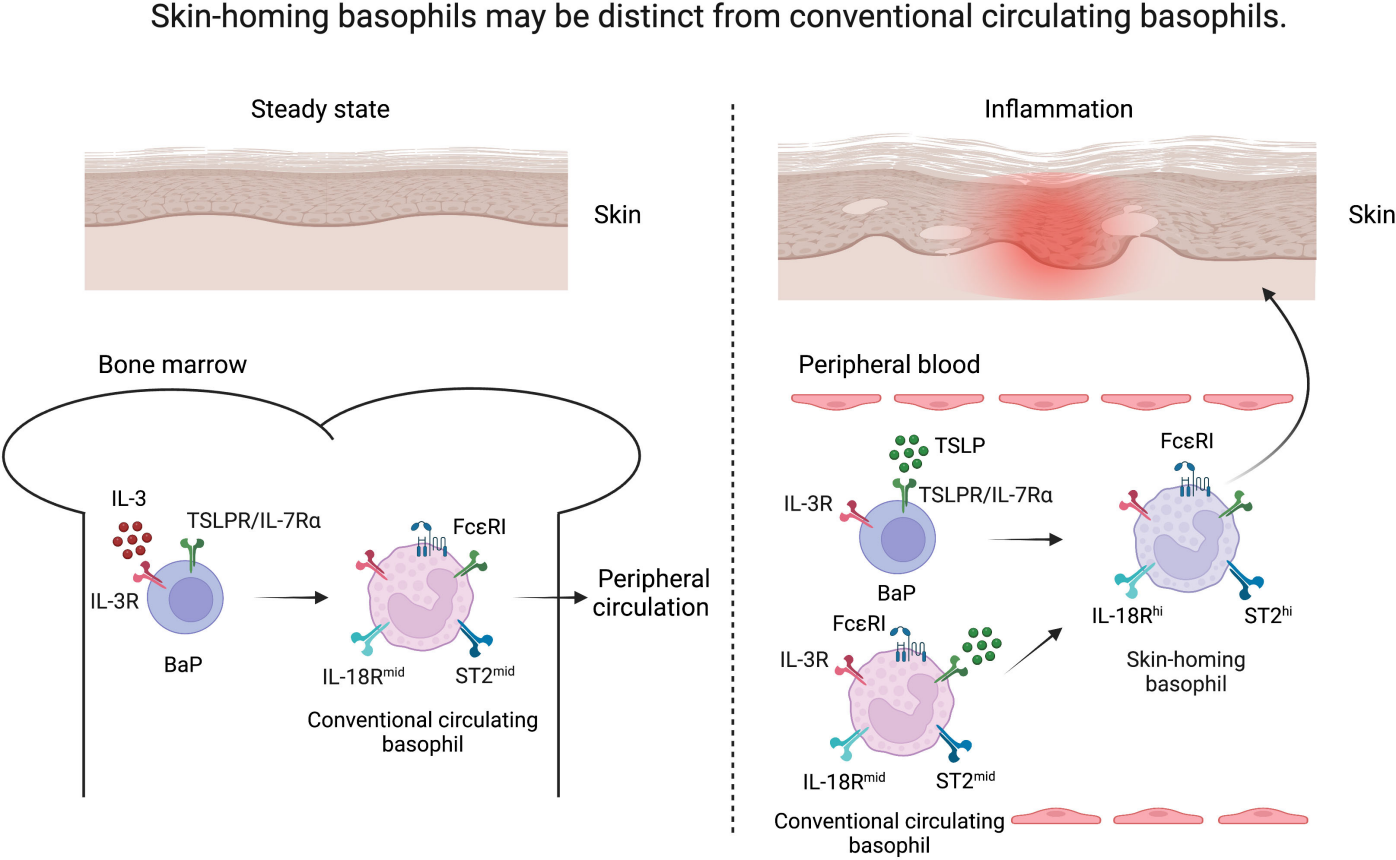
Skin-homing basophils may be distinct from conventional circulating basophils. Under steady state, basophil progenitors (BaP) develop into FcϵRI^hi^ basophils under the influence of IL-3 in the bone marrow (conventional circulating basophils, left). Upon inflammation, TSLP released from the skin drives the maturation of BaPs to exhibit a highly activated phenotype (skin-homing basophils, right) as evidenced by upregulation of IL-18R and ST2 in comparison to IL-3-elicited basophils.

Several studies have implicated TSLP-elicited basophils in murine models of allergic diseases such as AD, food allergy, and eosinophilic esophagitis (15, 16, 20). In addition, TSLP-elicited murine basophils exhibit a highly activated phenotype as evidenced by upregulation of key activating cytokine receptors including those for IL-18 (IL-18R) and IL-33 (ST2, IL-33R) in comparison to IL-3-elicited basophils (20). Both IL-18 and IL-33 are now considered canonical activating cytokines for basophils and strongly implicated in AD-associated inflammation in both mice and humans (44–48). These findings suggest that skin inflammation in AD may skew basophil development *via* epithelial cell-derived TSLP, creating a reservoir of basophils that can be rapidly activated by skin-associated IL-18 and IL-33. We refer to these basophils as uniquely skin-homing (**Figure 1**). Similar to IL-33, it is now appreciated that IL-18, in contrast to other organs, acts as an alarmin in the skin to potently promote type 2 immune responses (49). These findings may explain, in part, the failure of tezepelumab in phase 2 clinical trials for AD, as transient blockade of TSLP may not be sufficient to reset the population of basophils that are hyperresponsive to other alarmins in the skin (43). In other words, a typical 12-week clinical trial would likely not be able to capture clinical responses related to such biological effects. Notwithstanding the duration, another possibility is that TSLP blockade alone is no longer sufficient to suppress basophil-mediated skin inflammation after the accumulation of basophils in the skin that exhibit a unique transcriptional signature; such basophils may require simultaneous blockade of IL-18 and/or IL-33 for synergistic therapeutic efficacy. Future studies are warranted to determine the precise array of regulatory cytokines that need to be disrupted to suppress basophils and AD-associated inflammation.

## The emergence of skin-homing basophils

Classically considered short-lived, both murine and human basophils have been shown to rapidly lose their viability in a matter of a few days *in vivo* and *in vitro*, respectively (9, 50). However, these survival assays were performed on bulk populations of basophils from the bone marrow, blood, or spleen. It is increasingly appreciated that when basophils traffick into the skin (or possibly the lung), they can acquire distinct transcriptional and functional properties (21, 22). We have long observed that while basophils are generally absent in healthy skin, upon the induction of AD-like inflammation, they traffick into the skin as early as day 4 and persist stably through day 12, and likely well beyond (17, 51). Further, it has recently been shown that basophils in AD-like skin are distinct from splenic basophils and persist in the skin beyond the acute inflammatory phase to aid in the resolution of inflammation. Strikingly, these late-phase basophils promote the expansion of M2-like macrophages *via* cooperative production of IL-4 and monocyte colony-stimulating factor (M-CSF) (21). It has recently been shown that basophils which reside in the lung at early developmental stages imprint a unique developmental program in alveolar macrophages. Indeed, these lung-associated basophils demonstrated a distinct transcriptional profile from those in circulation (22). In addition to transcriptional differences, basophils in the skin could show morphologically distinct characteristics compared to circulating basophils. For example, Cheng et al. found that basophils accumulated in antigen-sensitized skin close to blood vessels, while those in non-sensitized skin were more widely distributed upon antigen challenge (52). Similarly, basophils have been shown to exhibit unique motility and apparent contacts with sensory neurons upon the antigen challenge as well in the setting of AD-like disease (53). Taken together, these studies provoke the hypothesis that basophils, upon entry into the skin, acquire a distinct transcriptional program leading to the distinct morphological changes and unique survival and effector programs not observed from traditional studies in the hematopoietic compartments, which likely focused more on conventional circulating basophils. However, it is important to note that these studies on basophil heterogeneity used *Mcpt8* as a basophil-specific marker for transcriptional studies and cell-depletion. Recent studies have suggested that integrinβ7^+^ mast cells also express Mcpt8 both in the skin and the lung under allergic inflammation (54, 55). Nevertheless, how basophils could acquire distinct identities in peripheral organs remains to be fully clarified and addressed.

While it is increasingly appreciated that there is developmental and functional heterogeneity of basophils, it has only recently come to light how diversity of basophil function can influence different aspects of a single disease (56, 57). For example, it is well-recognized that basophils are associated with human AD and promote the pathogenesis of AD-like disease in mice (17, 21, 23, 24, 27). By contrast, as described above, it has been observed that in the resolution phase of AD-like disease in mice, basophils in the skin also promote restoration of barrier function and disease resolution (21). In a context of itch sensation, basophils appear to be dispensable for chronic itch, while they are known to be essential for allergen-mediated acute itch (25, 27, 53). Indeed, it has been shown that TSLP promotes a program that is also highly enriched for the arachidonic acid pathway which leads to the production of leukotrienes and other bioactive lipids that serve as key effector molecules of murine basophils (20). One such leukotriene, namely LTC4 is now recognized as a very potent pruritogen (53, 58, 59). Taken together, these findings demonstrate the sophisticated array of effector functions orchestrated by basophils.

CSU exemplifies how skin-homing basophils can help to explain disease pathogenesis. CSU is an itchy, immune-mediated skin disorder that afflicts 1% of the population and has a profoundly negative impact on quality of life. It is defined by both hives and itch; these processes are mediated, in part, by activation of IgE and release of histamine from mast cells. Notwithstanding the role of mast cells, it is also appreciated that basophils accumulate in the lesions of urticaria, and that blood basophil deficiency is a feature of CSU (60). Thus, it is hypothesized that basophils recruited to the skin could contribute to the pathogenesis of CSU. The role of basophils in CSU is further suggested by a report that the number of basophils in the blood of CSU patients increases after anti-IgE mAb (omalizumab) treatment (60). Another study has revealed that the surface expression of FcϵRI on basophils was lower in CSU patients who showed better response to omalizumab (61). In addition, it is known that IgE and FcϵRI trigger the migration of murine mast cells toward antigens and that IgE and FcϵRI also mediate human basophil migration *in vitro* (62–64). Thus, these studies suggest that omalizumab may inhibit IgE-mediated activation in basophils, resulting in decreased motility into the skin. Future studies will be required to clarify this possibility.

However, the activation of basophils in CSU does not seem to be exclusively explained by IgE- and FcϵRI-mediated pathways. Antihistamines are the first-line therapy for CSU; however, even high doses are insufficient in 54% of patients (65). Anti-IgE therapy is the second-line strategy to which 40% of patients with CSU are refractory as well (66). These therapeutic gaps strongly suggest that other histamine- and IgE-independent pathways are operative. In 2015, a seminal paper by McNeil et al. identified that Mas-related G protein-coupled receptor B2 (MrgprB2), and its human ortholog, MRGPRX2 are key receptors that respond to a host of cationic neuropeptides and drugs that induce IgE-independent mast cell activation or allergic-like reactions (67). Indeed, MRGPRX2 has been identified as a possible biomarker in CSU (68). Although the expression and function of MRGPRX2 were mainly studied in mast cells, it has recently been reported that human basophils also express MRGPRX2 (69, 70). Given the potential role of MRGPRX2 on both mast cells and basophils, the heterogeneity of the therapeutic response in CSU may be explained, in part, by the overall composition of IgE-reactive vs. MRGPRX2-reactive mast cells and basophils, respectively, in CSU. This remains a major area of investigation to inform new pathways for treatment.

**Figure 2:**
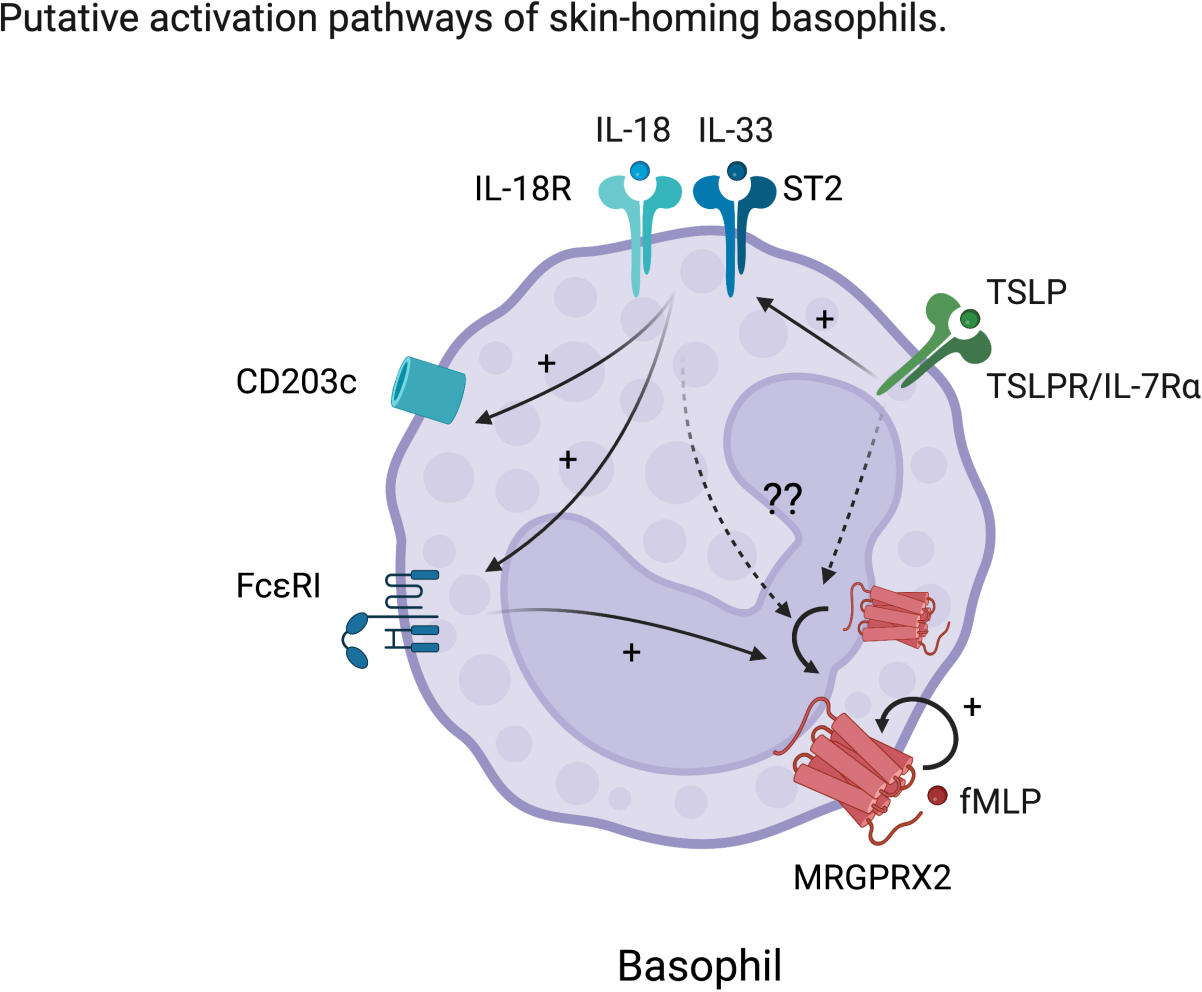
Hypothetic characteristic activation of skin-homing basophils. TSLP enhances the response to IL-18 and IL-33 in basophils. IL-18 and IL-33 further activate basophils resulting in upregulation of conventional activation markers such as CD203c and enhancement of FcϵRI expression. However, the effects of these skin-derived cues on MRGPRX2 expression in basophils are still unknown. We hypothesize that skin-derived cues also upregulate or externalize MRGPRX2, which is considered to be internalized at steady state on human basophils.

MRGPRX2 is now emerging as a therapeutic target in the field of allergy. However, MRGPRX2 expression on basophils either at steady state or upon activation remains a major area of controversy (71). It is hypothesized that MRGPRX2 is often internalized in basophils but could be exposed upon activation (71). In support of this, it has been shown that MRGPRX2 expression on human basophils was upregulated by cross-linking of IgE, complement component 5a (C5a), natural N-formyl peptide (fMLP) or IL-3 stimulation *in vitro* (69, 70). In relation to mast cells, MRGPRX2 function was promoted by TSLP but was dampened by SCF or IL-4 (72–74). Therefore, we speculate that maturation and/or activating factors for basophils including IL-18, IL-33, or TSLP could modulate MRGPRX2 expression on human basophils, contributing to their functional heterogeneity (**Figure 2**). To this end, future studies are required to determine the precise ligands and their effects on non-canonical basophil activation and function. We hypothesize that, given MRGPRX2’s close association with skin-resident mast cells, its expression on basophils likely marks their identity as also being more skin-associated or homing.

## Trafficking of basophils into the skin

Although we have discussed how immune dysregulation may promote the emergence of a unique population of basophils that is capable of responding to skin-derived signals, how basophils are recruited into the skin remains a mystery. It is well-known that basophils rapidly accumulate into peripheral tissues including the skin in a variety of settings such as helminth infection, tick bite, or allergic inflammation (11, 19, 75–77). However, there is still a paucity of evidence on the specific chemokines or cellular processes involved in basophil trafficking. Human studies *ex vivo* have revealed that basophils can migrate toward numerous chemokines (e.g., CCL2/3/5/7/11/13, and CXCL12/13), C5a, Prostaglandin D2 (PGD2), Thromboxane B2, urokinase-type plasminogen activator, and bacterial/viral peptides (fMLP and gp41) (78–87). Notably, serum levels of CCL2 were found to be elevated in a setting of venom- or food-induced anaphylaxis, which correlated a decrease in circulating basophil numbers (88). In addition, basophil accumulation in human skin or xenografted skin was observed after intradermal injection of CCL2 or CCL17, respectively (89, 90). Another study revealed increased migration of basophils in patients with systemic lupus erythematosus toward CXCL12 compared to those from healthy controls (91). A recent study by Blom et al. revealed that human basophils activated by IgE, C5a, or fMLP express various types of chemokine receptors including CCR4, CCR10, CCR6, CCR8, XCR1 and CCX-CKR, some of which are known as skin-homing receptors (6). In this study, they also found a bimodal expression of certain chemokine receptors such as XCR1, cutaneous lymphocyte antigen (CLA), or CXCR4 even among the CD63^+^ activated subset, further supporting phenotypic heterogeneity of human basophils upon activation. Puan et al. revealed that FUT6 is essential to sialyl-Lewis x (CD15s) expression on human basophils and its deficiency severely reduces their rolling capacity on E-selectins and cutaneous allergic symptoms (92). In mice, both PGD2 and CXCL12 have also been shown to be important in basophil trafficking to secondary lymphoid organs in a murine lupus model, while other studies showed CCL7-dependent migration to the draining lymph nodes in a context of pancreatic tumor or type 2 skin inflammation (91, 93–95). The accumulation of basophils in the lymph nodes after helminth infection depends on IL-3 from CD4^+^ T cells (96), while IL-3 supplied by skin-resident CD4^+^ memory T cells is essential for their recruitment to the skin in the setting of tick bite (97). In the setting of AD-associated inflammation, basophil recruitment to the skin is uniquely dependent on TSLP (20); similar dependence on TSLP has also been observed with intradermal injection of lipoteichoic acid (LTA), a cell wall component of bacteria (98). Moreover, under TPA-induced chronic skin inflammation, TSLP and IL-3 externalize CXCR4 expression on basophils and their recruitment to the skin depends on CXCL12-CXCR4 axis and IgE (99). Taken together, these studies demonstrate that a number of factors have been implicated in basophil trafficking in the past (**Table 1**). However, future studies will have to be aimed at understanding the tissue-specific signals that drive basophil migration into various organs and their unique interactions in the context of disease.

**Table 1:** *In vivo* or *ex vivo* evidence for basophil trafficking to tissues.

Species	Trafficking sites	Factor	Tentative Source	Experimental or disease condition	Experiment type	Ref
Human	N/A	CCL2, CCL5, CCL7	N/A	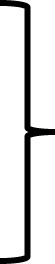 Transwell migiration	*Ex vivo*	(78)
		CCL5, CCL7, CCL11, CCL13			*Ex vivo*	(79)
		CCL2, CCL3, CCL5, CCL11, CXCL12			*Ex vivo*	(80)
		CCL2, CCL11, CXCL12, IL-8			*Ex vivo*	(81)
		CCL2, CCL11		Transendothelial migration	*Ex vivo*	(82)
		C5a		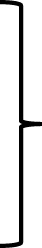 Transwell migiration	*Ex vivo*	(83)
		Prostaglandin D2			*Ex vivo*	(84)
		Thromboxane B2			*Ex vivo*	(85)
		Urokinase-type plasminogen activator			*Ex vivo*	(86)
		fMLP or gp41			*Ex vivo*	(87)
		CD15s		Rolling assay	*Ex vivo*	(92)
Human	N/A	CCL2	Stromal cells?	Anaphylaxis	*In vivo*	(88)
	Skin	CCL2	N/A	Intradermal injection into human skin	*In vivo*	(89)
	Skin	CCL17	Endothelial cells, Keratinocytes?	Intradermal injection into skin xenograft of humanized mice	*In vivo*	(90)
	Secondary lymphoid organs	CXCL12	N/A	Systemic lupus erythematosus	*In vivo*	(91)
	Skin?	CD15s	Basphils	Mosquito-bite or skin prick test to house dust mite	*In vivo*	(92)
Murine	Skin	IL-3	CD4+ T cells	Tick-bite	*In vivo*	(97)
	Skin	TSLP	N/A	MC903-induced skin inflammation	*In vivo*	(20)
	Skin	TSLP	Keratinocytes?	Lipoteichoic acid injection	*In vivo*	(98)
	Skin	TSLP/IL-3, CXCR4 and IgE	N/A	TPA-induced skin inflammation	*In vivo*	(99)
	Lymph nodes	IL-3	CD4+ T cells	Helminth infection	*In vivo*	(96)
	Lymph nodes	PGD2	N/A	Lupus nephritis	*In vivo*	(91)
	Lymph nodes	CXCL12	N/A	Lupus nephritis	*In vivo*	(93)
	Lymph nodes	CCL7	Monocytes	Pancreatic cancer	*Ex vivo*	(94)
	Lymph nodes	CCL7	Dendritic cells	Papain-induced type 2 skin inflammation	*In vivo*	(95)

N/A, not applicable.

Additionally, it appears that basophils can exhibit heterogeneous behavior even within the same tissue under the same inflammatory condition. We have found that *in vivo* stimulation with allergen in the skin results in the emergence of two distinct populations of basophils - one that is enlarged and immotile and another that is small and highly motile in the setting of AD-associated acute itch flares (53). Although why such heterogeneity of motility exists within the skin remains unknown, these findings support the hypothesis that there are likely numerous different subsets of basophils across tissues that respond differentially to even the same signals. Thus, basophil trafficking could be regulated in a subset-dependent manner, indicating increasing complexity in terms of their regulation.

As noted above, there is significant evidence that basophils imprint unique transcriptional and functional programs onto macrophages in the skin and lung (21, 22). However, whether skin-resident macrophages recruit basophils into the skin *via* reciprocal interactions remain to be shown. There is a large body of work that suggests that other circulating granulocytes like neutrophils are heavily influenced by tissue-resident macrophage-derived signals upon tissue damage or pathogen entry (100–102). Indeed, macrophages are capable of producing various types of mediators which have been implicated in basophil chemotaxis *in vitro* (e.g., CCL2, CXCL1, CXCL2, C5a) (103, 104). Thus, we speculate that homologous mechanisms likely underlie basophil recruitment as well in the context of helminth parasite invasion or allergic barrier disruption. Future studies will be required to determine the full range of cellular and molecular cues that aid in the homing of basophils into the skin.

Finally, whether specific populations of basophils go back into the circulation and travel distally also remains poorly understood. In the setting of helminth infection, it is reported that group 2 innate lymphoid cells (ILC2s) in the tissue are extruded to the circulation to disseminate type 2 inflammation (105). Both skin-homing basophils and ILC2s receive similar activation cues from the skin (e.g., IL-18 or IL-33) to critically mediate type 2 inflammation, despite being rare populations. In light of our speculation that skin-homing basophils acquire the ability to survive much longer, it is possible that basophils could also move from the skin into the circulation and on to other distal sites. However, future studies will be required to fully understand the importance of basophil movement into and out of the skin.

## Conclusion

The unique characteristics of basophils have been greatly informed in the last decade due the development of unique tools. Studies using animal models have revealed their critical involvement in a number of disease states in the skin including helminth infection, tick bites, and AD (15–17, 106). However, in addition to their ability to promote allergy, basophils are increasingly appreciated for their dynamic ability to respond to allergen, cytokines, and exhibit both proinflammatory and restorative properties. By understanding how specific subsets of basophils may have unique proinflammatory, survival, and survival properties, we speculate that selectively targeting such basophils may represent a highly effective therapeutic strategy for a variety of skin diseases such as AD, or CSU.

## Data availability statement

The original contributions presented in the study are included in the article/supplementary material. Further inquiries can be directed to the corresponding author.

## Author contributions

RS contributed to writing manuscript and crating Figures under supervision of BK. All authors contributed to the article and approved the submitted version.
